# Walking Behavior of Older Adults in Temuco, Chile: The Contribution of the Built Environment and Socio-Demographic Factors

**DOI:** 10.3390/ijerph192214625

**Published:** 2022-11-08

**Authors:** Mohammad Paydar, Asal Kamani Fard

**Affiliations:** 1 Escuela de Arquitectura Temuco, Facultad de Ciencias Sociales y Artes, Universidad Mayor, Av. Alemania 0281, Temuco 4780000, Chile; 2 Universidad Católica del Maule, San Miguel 3605, Talca 3460000, Chile

**Keywords:** walking behavior, older adults, socio-demographic factors, built environment

## Abstract

The amount of walking for daily transport has decreased significantly over the last decades in Temuco, Chile. Moreover, the percentage of older adults (aged over 65) who did not meet the recommendations of at least 150 min of physical activity per week has increased during this time. In this regard, the present study examines the contribution of socio-demographic and built environment factors on the walking behavior of older adults in Temuco, Chile, with a view to improving their level of physical activity. A cross-sectional study was conducted among 463 older adults aged 60 years and over. Travel Diary Data taken from “Encuesta Origin Destino” (EOD) 2013, Geographic information systems (GIS), audits (PEDS with certain revisions) and finally, multiple regression analysis, were used to examine the objectives. Associations were found between the walking behavior of older adults and several socio-demographic factors, as well as several built environment factors including destination (the number of parks and the land use mix), functionality (street connectivity, length of street sections and off-street parking lots) and aesthetics (views of nature, building height, and articulation in building design). These findings should be considered by urban/transport policymakers to improve the walking behavior of older adults in this city.

## 1. Introduction

Walking is one of the most sustainable modes of transport due to its accessibility, lower levels of pollution, and lower cost [1]. An increase in the amount of walking for daily transport contributes to maintaining the minimum rate of physical activity and therefore has a positive impact on public health [2]. The need for an increase in walking for utilitarian purposes was also emphasized during the recent COVID-19 pandemic [3,4]. More than 150 cities have expanded emergency walking infrastructure as of late April 2020 to increase their resilience in the context of the COVID-19 pandemic [5]. This pandemic has also fostered a faster adoption of sustainable mobility measures including street pedestrianization in Latin American cities [6]. Although there is a growing interest among Latin American researchers in walking behavior [7,8,9,10], most of the literature addressing the built environment and walking is produced was Europe and the United States [9]. With respect to Chile, the few existing studies on the improvement of walking behavior and its contributing factors are focused on Santiago, the capital, which differs significantly in terms of its structure and size from the rest of the cities in Chile [9,11]. For instance, the rate of walking decreased (by 5%) in daily transport trips from 2003 to 2013 in Temuco, which is one of the medium-sized cities in southern Chile [12]. This shows the need to increase the amount of walking for utilitarian purposes in this city. Moreover, the percentage of older adults (aged above 65) who did not fulfill the recommendations of at least 150 min of physical activity per week has increased by 9% from 2010 to 2017 in Chile [13,14]. This emphasizes the importance of improving the physical activity and walking behavior of older adults in this country. In this regard, increasing the amount of walking for daily transport among older adults makes an important contribution toward meeting their recommended level of physical activity.

According to ecological models, walking behavior is influenced by a complex relationship between environmental (physical and social) and individual characteristics [15,16,17]. In this regard, previous studies—that were mostly carried out in developed countries—demonstrated the role of several personal, socio-demographic, social and built environment factors, as well as accessibility and walking amenities for improving walking behavior among older adults [18,19,20]. In particular, regarding socio-demographic factors, gender, age, and education were found to be related to the walking behavior of older adults [16,21,22,23,24,25]. For instance, old men walk significantly more than old women in Chicago, United States [23]. However, Krogstad et al. (2015) found that women walk more than men when running errands or meeting with others in Kristiansand, Norway [16]. Other studies found relationships between the walking behavior of older adults and their job, income, marital status, number of vehicles, and Body Mass Index (BMI) as well [26,27,28]. Van Cauwenberg et al. [29] also demonstrated the association between familiarity with the walking environment and the walking behavior of older adults in Belgium.

Furthermore, the built environment plays an important role in improving the walking behavior of older adults since they are more sensitive than other groups to the built environment’s suitability for walking [30]. Increasing the population/housing density contributes to improving the walking habits of older adults [31,32,33]. Access to different facilities such as retail outlets and restaurants as well as recreational spaces such as parks and playgrounds, all contribute to increased walking among older adults in Singapore, England, and the United States [34,35,36,37,38,39]. The positive effects of access to parks and green spaces on the walking behavior of older adults were also supported by studies in Latin American countries such as Brazil and Chile [40,41,42]. In addition, mixed land use is positively correlated with the walking behavior of older adults in Canada, China and Hong Kong [43,44,45]. However, Thornton et al. (2017) found a negative correlation between mixed land use and walking for recreation among elderly people in the United States [36].

Infrastructure or the functional aspects of walking environments, also have an impact on the walking behavior of older adults. For instance, a more interconnected walking network [19,36,37,46], the presence of sidewalks and the quality of the pavement [31,40,45,47], a greater width of sidewalks and pathways [48], amenities along the sidewalks including facilities for resting/sitting [43,49,50] and less physical barriers along the sidewalks [51], all have an effect on the walking behavior of older adults. Moreover, safety from traffic and personal security is associated with the walking behavior of older adults in the United States and Belgium [29,52,53]. This is supported by studies in developing countries such as Brazil, Colombia, and Nigeria which found a relationship between the walking behavior of older adults and features relating to traffic safety and personal security [40,42,54,55]. For instance, the presence of crosswalks [40,52] and traffic control devices such as pedestrian signals [29,52], lead to increased walking in older adults. Less traffic volume also leads to a higher tendency to walk among older adults in Hong Kong [56]. The environmental features that relate to personal security and have an impact on the walking habits of older adults include street lighting [40,57], the presence of other people along the walkways [29,58], signs of disorders, physical incivilities, and stray animals [23,57,59]. A greater level of surveillance from adjacent buildings including a lower percentage of blind walls also contributes to improving the walking behavior of older adults [60,61].

Furthermore, more attractive and aesthetic walking environments contribute to improving the walking behavior of older adults [32,62,63,64]. The presence of natural features positively influences the walking behavior of older adults in Hong Kong [57]. Litter along the sidewalks had a negative correlation with the walking of older adults in the United States [52]. The walking behavior of older adults is also improved by the presence of parks and green spaces [39,61,62,63]. The number of trees along the walkways, front gardens, and the amount of green areas also contribute to encouraging walking in older adults in the Netherlands [61,65]. In addition, the type of building façades and their level of maintenance can influence the walking behavior of older adults [29,60]. Finally, the height of the buildings and the level of enclosure all have an impact on older people’s walking habits [65].

As it was reviewed, most of the studies which address the association between the walking behavior of older adults and the built environment were carried out in developed countries and there is a lack of the studies on walking behavior of older adults in Latin America and Chile, specifically the medium-sized cities of Chile such as Temuco. This research aims to examine the association between the socio-demographic as well as built environment factors and the walking behavior of older adults in Temuco, Chile. This would help the urban and transport policymakers to improve the walking behavior of older adults in this city and in other similar contexts in Chile. The research questions of this study are as follows:

What socio-demographic and built environment factors are associated with the walking behavior of older adults in Temuco, Chile?

What is/are the major difference/s between the current context and developed countries, regarding the factors that are associated with walking behavior among older adults?

## 2. Materials and Methods

### 2.1. Study Population

Temuco, the capital of the Araucania region, is one of the medium-sized southern cities with a population of about three hundred thousand people according to the 2017 Census. According to the 2017 census, 12.6% of the population of Temuco (about 38,000 people) are considered older adults (more than 60 years old) [66]. According to the 2017 National Health Survey (ENS) [13], the prevalence of individuals (older adults) who did not attain the recommendations of at least 150 min of physical activity, either through leisure activities or transport, was found to be 77% of those studied. Based on this information, to calculate the sample size, the following equation was used for estimating the proportions [67]. This equation was also used by previous studies on walking behavior in Latin American countries such as Brazil [42].

n0 = [P(1 − P)/(d/z)2].deff

The values applied to it were z = 1.96 (the value on a reduced normal curve corresponding to a confidence level of 95%), d = 0.065 (the sampling error accepted), deff = 1.54 (the design effect), and P = 0.23 (the proportion of individuals to be estimated for engagement in physical activities). By applying these values in the formula, the sample size was calculated to be a minimum of 410 older individuals. However, due to the limitations during the pandemic in terms of face-to-face contact (this research was carried out in 2021), the results of the travel diary data taken from “Encuesta Origin Destino (EOD), Hogar y Viajes”, carried out by the ministry of transport for Chile were considered [12]. According to this survey, the total number of older adults who walk during their daily trips is 463 respondents, which covers the minimum number of older adults calculated for this study. Accordingly, parts of the required data including the walking behavior of older adults and the socio-demographic factors were extracted from the EOD survey, which is further explained in the next sections.

### 2.2. Measurements

#### 2.2.1. Transport Walking and Socio-Demographic Variables

**Figure 1:**
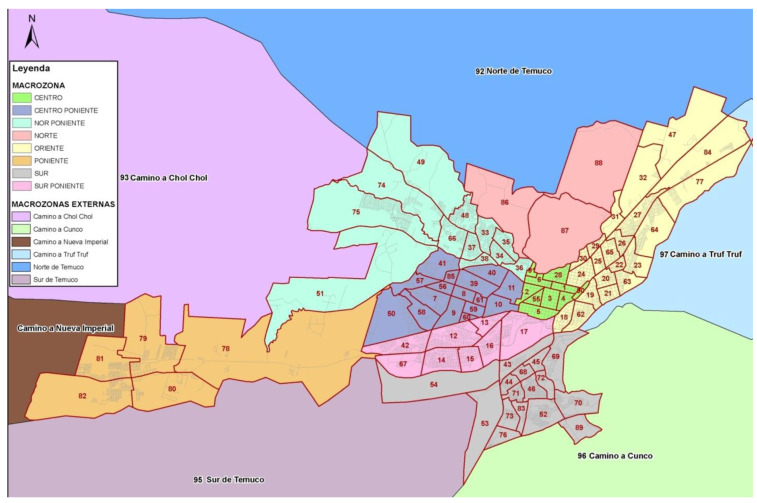
Zoning defined in Stage I of the travel diary study in Temuco (Taken from Actualización Plan De Transporte Temuco, Etapa II [12].

The information of Travel Diary Data taken from “Encuesta Origin Destino (EOD), Hogar y Viajes”, performed by the ministry of transport, Chile, Temuco 2013, was used to measure walking behavior and the relevant socio-demographic factors [12]. EOD is a general-purpose survey covering a wide range of issues including transport and travel. Participants completed a travel diary that detailed all journeys undertaken on the day of travel registration. For each journey stage, data collected included the origin, destination, purpose (assigned to all stages that comprised a given journey), distance, and mode of travel. In addition, the EOD survey registers the socio-demographic characteristics of the respondents and the households to which they belong. In other words, it includes everything necessary to evaluate travel behavior patterns and the socioeconomic characteristics of travelers [64]. The urban transport zone map of Temuco, which covers the entire area within the urban limit, was used as the basis for the travel diary study in this city. The data collection of EOD was carried out based on these zones. The zones consist of 8 macro sectors (with different colors) and 91 sectors (with numbers) within the macro sectors (Figure 1). The hierarchy of the urban transport network, the homogeneity of land use, socio-economic characteristics of the inhabitants and natural barriers were some of the criteria to be considered for defining the different zones.

According to EOD 2013, 1721 people used walking for daily travel, and among them, 463 older adults (age above 60) were identified. A travel diary survey was applied by previous studies to measure walking behavior and its relevant socio-demographic factors as well [68,69,70]. Walking behavior could be measured for each participant if walking was included in his/her travel diary since walking was an attribute assigned to each journey stage (minutes of walking dedicated to each trip). Accordingly, the total minutes of walking, taken from all the reported walking trips of each respondent was calculated as the dependent variable.

EOD Home and Travel also provided the required information for socio-demographic variables such as household size, workers per household, adults per household, income (household or individual), number of vehicles in each household, possession of driver’s license, gender, employment status, and age. A continuous variable such as income was transformed into a categorical variable including two categories of low versus middle income since most of the respondents fall into these two categories. In addition, duration of living was used as the indicator of familiarity. It was measured through two categories of less than one year versus more than one year of living in the house, which could be a better representative of a low versus a medium level of familiarity with the walking environment. Finally, education was divided into three categories: low-level, intermediate-level, or high-level education.

#### 2.2.2. Built Environment Variables

The previous studies in transport that focused on walking behavior have classified the built environment factors relating to walking in a “3D” model including Density, Diversity, and Design. Later, it became “5D” including Density, Diversity, Design, Destination accessibility, and Distance to transit [71]. According to Pikora et al., (2003) [72], the main built environment factors that relate to active travel including walking and cycling are Functional, Safety, Aesthetic, and Destination. In this study, the following classifications regarding built environment factors (domains) were used: Density; Destinations; Functional and infrastructure-related factors; Traffic Safety; Personal Security; and Aesthetics (Table 1). This classification covers most of the built environment factors which influence the walking behavior of older adults according to previous studies. The factors of each group were measured objectively using Geographic Information Systems (GIS) tools and/or Audit (PEDS mixed with certain items of SPACES) depending on the nature of the factor whether it was a micro-scale or macro-scale factor related to the built environment and considering how the previous studies normally measure each factor (Table 1). For instance, previous studies have shown that the macro-scale measurements of built environments relating to walking are more reliable—while measuring through GIS—than audit instruments [63,73]. These measurements are mostly related to density and accessibility in terms of the presence of destinations, land use mix, and street connectivity [63,73,74,75]. Thus, GIS was used to measure these factors in the selected buffer zones. The summary measures in regard to the built environment—by both GIS and audit instruments—are shown in Table 1. These factors were measured in the buffer zones with a radius of 400 m around the household in EOD which undertook the walking trips. The buffer size is a generally accepted “walkable” distance in existing research and could capture attributes of built environments immediate to one’s residence [71].

Concerning the built environment factors, measured through GIS, density was measured through both population density (number of inhabitants in each zone) and housing density (number of housing units in each buffer). Some information in relation to population density was also taken from National Census (CENSO 2017) [66]. The entropy index was used to measure land-use diversity which includes five main types of land use in this city: residential, commercial, services, and educational and health centers as well as hospitals. The numbers of each type of land use in each buffer were also measured to calculate destination accessibility. Access to public transport was calculated by measuring the total street length with access to public transport per area of each buffer zone. Connectivity was measured using three indicators of Link–Node Ratio (links per unit of area (streets)/# Nodes per unit of area); Intersection Density (real nodes area / area) and Street density (total street length per unit of area/area) [76]. The literature suggests that a link–node ratio of 1.4 or 1.2 indicates connected networks. Finally, the slope was measured using three values: “high slope” where most streets of the buffer have more than 15% slope, “medium slope” where most streets of the buffer have between 5% to 15% slope, and “low slope” which is less than 5% slope.

With respect to street audit, firstly, different audit tools—developed in different contexts around the world—were considered including The Irvine–Minnesota Inventory (I–M), The Walking Suitability Assessment Form (WSAF), Pedestrian Environmental Data Scan (PEDS), Systematic Pedestrian and Cycling Environmental Scan (SPACES), The Walkable Places Survey (WPS) and Analytic Audit Tool. By comparing and adjusting the previously mentioned audit tools within this context, it was found that Pedestrian Environment Data Scan (PEDS) is a more appropriate audit tool to measure built environment factors of sidewalk environments in our context [77]. Thus, PEDS was used as the basis for measuring the built environment factors. Inter and intra-rater reliability of items in the instrument has previously been found to be high [77]. However, PEDS was modified by adding five items taken from the Systematic Pedestrian and Cycling Environmental Scan (SPACES) due to certain requirements in this context. For instance, “Surveillance (visibility) from the windows” and “Type of Views” were taken from SPACES and added to PEDS. Inter and intra-rater reliability of items in the instrument of SPACES has previously been found to be high as well [72]. The final audit instrument consisted of forty-five items. Within a 400 m radius, trained auditors carried out an objective environmental audit on each segment—both sides—by filling in a modified version of the Pedestrian Environment Data Scan (PEDS) as explained previously. A reliability audit was conducted where two field auditors re-audited the segments of each zone. All the items, except six—which showed low inter-rater reliability (kappa < 0.40)—had moderate to high inter-rater reliability (kappa > 0.40). The items with low inter-rater reliability were excluded from the summary environmental measures. Examples of these items are “The level of front yard gardens”, “the percentage of blind walls in the segment”, and “number of abandoned buildings in the segment”. The final thirty-nine remaining items—used for summary measures (Table 1)—are related to the functionality including the design of the walkway’s structural features (10 items), design of the street’s structural features (4 items), design related to permeability (street connectivity) (1 item), safety, including traffic safety (5 items), personal security (3 items), aesthetics including streetscape (7 items), views (3 items), and finally destinations including the presence of different types of destinations (5 items), and land use mix (1 item).

### 2.3. Analysis

SPSS software version 23.0 was used to analyze the data. LOESS (locally weighted smoothing) was used to examine continuous measures of built environment. This aids in the selection of inflection points to categorize built-environment variables. To predict a dependent variable from the independent variables, hierarchical multiple regression analysis was applied. Firstly, the model that contained variables relating to the socio-demographic attributes of respondents and their households was used. In the next step, the built environment factors were added. During this step, firstly, the built environment factors including “Roadway/path lighting”, “Presence of medium-high volume driveways”, “Presence of public art”, and “Presence of houses”—that did not show variability or very little variability along the buffer zones—were excluded from further analysis. Then, the variables which showed high multi-collinearity (VIF > 5) were deleted. In this way, the final model represents the combination of socio-demographic variables and representations of the built environment factors that were interpretable, minimally inter-correlated, and consistent with the theory (Table 1).

## 3. Results

### 3.1. Sample Statistics

The descriptive statistics of the socio-demographic variables and familiarity are shown in Table 2. Most of the respondents are female (63.5%) as compared to male (36.5%) and the majority of the respondents are the owners of their houses (85.5%). Added to this, the majority of respondents do not have a driver’s license (76.4%) and most of them do not have private cars in their households (64.4%). Most respondents have an educational level of secondary school or below (86.8%). Furthermore, the average number of family members is 3.08 persons in each household and most of the respondents have a high familiarity with the walking environment since most of them have lived for more than 1 year in their current home (88.3%). Finally, 15.87 min of walking per day is the mean of the walking level of the total respondents.

### 3.2. The Factors Associated with the Walking Behavior of Older Adults

Table 3 shows the results of the regression analysis between the walking behavior of older adults and its contributing factors. The variables in Table 3 explain 24% of the variance in older adults’ walking behavior (R^2^ = 0.240). Men walk significantly more than women (β = 0.156, *p* = 0.005) and more elderly older adults walk significantly more as well (β = 0.127, *p* = 0.032). In addition, the “Number of total trips in each household” was shown to have a significant negative correlation with walking (β = −0.151, *p* = 0.037).

Regarding the built environment factors, a higher number of parks and plazas is associated with improving the walking behavior of older adults (β = 0.164, *p* = 0.016). In contrast, a higher number of educational destinations—leading to greater accessibility to this type of destination—contributes to less walking among older adults (β = −0.155, *p* = 0.017). Mixed land use showed a significant positive correlation with the walking level of older adults (β = 0.148, *p* = 0.040) which shows that a higher diversity of land use contributes to increased walking among older adults. From the functional aspects, a greater length of walkways is associated with encouraging walking among older adults (β = 0.140, *p* = 0.029). More off-street parking lot spaces contribute to a decrease in the walking behavior of older adults and vice versa (β = −0.185, *p* = 0.008). In addition, a higher link–node ratio, which is one of the indicators of network connectivity, is related to less walking among older adults and vice versa (β = −0.162, *p* = 0.022). Finally, from the aesthetic-related features, more frequent views of nature including parks and community gardens, greater building height, and higher articulation in building design are associated with increasing the level of walking in older adults (β = 0.159, *p* = 0.012; β = 0.166, *p* = 0.033; β = 0.116, *p* = 0.052).

## 4. Discussion

The first research question of this study is what socio-demographic and built environment factors are associated with the walking behavior of older adults in Temuco, Chile?

In terms of the association between the socio-demographic factors and walking behavior of older adults, men walk significantly more compared to women in this city. Previous studies support the impact of gender on the walking behavior of older adults [16,23]. However, while Mendes de Leon et al. (2009) [23] found that men walk significantly more than women (among older adults), Krogstad et al. (2015) [16] found that women walk more than men when running errands or meeting with others. One interpretation is that women are generally more vulnerable than men regarding potential threats that may occur during walking [78,79]. In addition, among older adults, those of greater age are considerably more likely to walk in this city. This is in contrast with the results of previous studies which found that physical activity, as well as walking behavior among older adults, reduces with age [23,80].

It was also found that most of the older adults who walk in this city do not have a private car or a driver’s license. Thus, private cars generally have no role in the lives of older adults who walk in this city. These results also show an incompatibility between walking and using private cars for daily transport among older adults who walk in this city. This inference is reinforced while understanding that the “Number of trips in each household” was shown to have a significant negative correlation with walking. Increasing other types of travel modes used for daily trips among older adults contribute to a decrease in their walking behavior. However, the current research did not address other modes of transportation such as private cars; and this initial inference needs further investigation by future studies.

Additionally, most of the older adults who walk in this context are low-income adults. This follows the main walking pattern in Chile and even Latin America in which most of the people who walk are from low-income families [8]. This is while in several developed countries a considerable percentage of the older adults who walk belong to high socio-economic status as well [37,53,81]. This finding partially responds to the second research question with respect to the difference between this context and developed countries in terms of the contributing factors to the walking behavior of older adults.

Several built environmental factors were also found to be associated with the walking behavior of older adults. Mixed land use is associated with improving the walking behavior of older adults in this city. This finding is supported by previous studies which found a positive correlation between mixed land use and the walking behavior of older adults [36,43,73,82,83]. Furthermore, a higher number of parks and plazas is related to improving the walking behavior of older adults in this context. This is supported by previous studies which have shown that access to recreational spaces such as parks and playgrounds as well as other recreational facilities, increases walking among older adults [36,37,40,81]. These studies stressed the importance of the recreational aspects of parks and their facilities for improving the walking behavior of older adults. Similarly, views of nature including public green spaces—one of the aesthetic-related features—are associated with encouraging walking in older adults of this city. This finding is also supported by previous studies, which showed the importance of aesthetic factors such as the presence of green spaces and parks for encouraging walking among older adults [39,62,63,83,84,85]. However, these studies showed the impact of recreational green spaces on recreational walking rather than transport walking [86]. These findings show that aspects of parks and plazas, such as the aesthetics of green spaces and the provision of recreational areas with public facilities are important for improving the walking behavior of older adults in this city. It should also be considered that Temuco suffers from a lack of parks and plazas in its urban sectors and that the parks and plazas it has are not in very good condition [12]. Although a large urban park has recently been opened in Temuco, there is no good access to this park for many urban sectors, especially for older adults. This finding is therefore applicable to the city’s urban and transport policy makers; they should raise the quality and quantity of recreational areas and green spaces such as parks and plazas in the different urban sectors to improve the walking behavior of older adults in this city.

In addition, regarding the aesthetic-related features, a higher degree of articulation in building design and greater building height both encourage walking in older adults of this city. Articulation in building design which refers to the degree of variety in buildings’ facades is one of the important indicators of aesthetic walking environments. This result is in line with previous studies, which have found a correlation between higher walking levels in older adults and a higher degree of variety in buildings’ façades [51,62,64]. Previous findings have also shown the relationship between the height of the buildings and the degree of walkway enclosure with the walking behavior of older adults [65]. Temuco is traditionally a flat city with one or two-story buildings in most urban sectors except for the center of the city. However, the recent approach toward a more compact city with the construction of high-rise buildings was observed within the last two decades in this city, especially in the center of the city and its surrounding urban sectors. This finding shows that the trend towards higher buildings—which provides a higher degree of enclosure—contributes to encouraging walking in older adults. Thus, the approach toward higher buildings and a more compact city is to be reinforced by urban/transport policymakers of Temuco in order to improve the walking level and public health of older adults in this city.

From among the functional aspects, more off-street parking lots are associated with a decrease in walking among older adults in this city and vice versa. The association between this feature and the walking behavior of older adults could be due to different functional and aesthetic aspects. For instance, more off-street parking lot spaces may decrease the sense of safety for older adults. In addition, more off-street parking lot spaces may also reduce the attractiveness of the views for older adults, leading to less walking. The reason/s for the emergence of such findings could be further investigated by future studies. Moreover, off-street parking lot spaces have increased within the last decades in Temuco due to a higher number of malls and a greater level of commercial land use in this city. Future commercial development, therefore, needs to consider this finding and try two things: firstly, include such off-street parking spaces inside new commercial buildings and secondly, design any outdoor off-street parking spaces in such a way to minimize interactions with pedestrians and improve the walking behavior of older adults.

Finally, a higher link–node ratio, which is one of the indicators of network connectivity, is related to less walking among older adults in this city and vice versa. Older adults walk more when there is a more disconnected urban street pattern. This is in contrast to the results of previous studies which found that a better-connected street network is associated with a higher rate of walking among older adults [19,31,34,36,46]. Although most of these studies were carried out in developed countries, a study in Colombia (South America) found a similar result in terms of the correlation between street connectivity and walking behavior among older adults [54]. This result could be considered in the light of the crime level and sense of insecurity, whose correlation to overall walking, in a similar environment, was also found by previous studies [41]. The number of gated communities within residential neighborhoods has increased within the last decade in different cities of this country in order to enhance actual security as well as the sense of security among the residents. Gated communities are usually residential areas restricted by fences and walls. The actual crime rate as well as fear of crime create situations where the inhabitants prefer to walk more in disconnected street patterns than connected ones. This situation regarding the design and layout of streets in relation to walking behavior could also describe one of the major differences between this context and the developed countries in terms of the association between the factors of the built environment and the walking behavior of older adults.

## 5. Strengths and Limitations

The main strength of this study is that it covers most of the built environmental factors examined by previous studies in regard to the walking behavior of older adults. In addition, using the instruments of GIS and audit meant that the built environment could be measured through different micro-scale and/or macro-scale entities, as mentioned by previous studies. Due to the limitation of COVID-19 in terms of access to people and implementing research in the field during the year 2021, it was decided to use the information from the EOD survey of 2013 as a part of the data of this research project. EOD 2013 is the latest survey regarding daily transport, implemented by the Ministry of Transport and Telecommunications, in Temuco, Chile. In this regard, the limitations of the study are the cross-sectional design and the measurement of walking behavior through a self-reported questionnaire. Self-report measurement requires the good memory of the participants and high estimation skills. Consequently, measurement errors may exist due to a lack of valid recall [87]. Another limitation of the study is the use of a long questionnaire to measure travel mode choice and the other variables, which may result in nonresponse and invalid results [24]. Furthermore, this study used objective measurements of the built environment factors. The addition of more subjective or perceptual aspects of the built environment may lead to a better understanding of the correlation between the built environment and the walking behavior of older adults in this city. Finally, it is to be noted that besides the studied factors in this research, there are also other important relevant factors to the walking behavior of older adults—such as the health status of older adults and the social related factors—which were not addressed here. The association between these factors and the walking behavior of older adults could be addressed by future studies.

## 6. Conclusions

While private car use has increased as a means of daily transport in recent decades in Temuco, the amount of walking has shown a notable decline in this mid-sized city of southern Chile. Most of the older adults who walk in this city do not have driving licenses or private cars in their households, which means that they do not tend to use private cars as a mode of transport. Regarding the first research question, the impact of several socio-demographic factors on walking behavior was found in this study including age, gender and the number of total trips in each household. The more elderly older adults walk significantly more, which is in general contrast to the findings of studies in other parts of the world. The policymakers of this city should pay attention to these socio-demographic factors to improve the walking behavior of older adults in this city.

In addition, the association between several built environment factors and the walking level of older adults was found, including the length of street sections, off-street parking lot spaces, building height, articulation in building design, and land use mix. These features are related to functionality, aesthetics, and destination. These findings were discussed and their implications were presented so that they could be used by urban/transport policymakers to encourage walking among older adults in Temuco and improve their health. One of the significant results concerning the built environment is that a higher number of parks and plazas leads to increased walking among older adults. Temuco suffers from poor quality and quantity of recreational public spaces such as parks and plazas in its different urban sectors. Special attention is needed on the part of the city’s urban/transport policymakers in order to address this weakness.

Furthermore, one of the important implications of this study is regarding the relationship between street connectivity and the walking behavior of older adults. Greater network connectivity was shown to have a negative correlation with the amount of walking among older adults, which means that older adults tend to walk more in disconnected urban sectors within this city. This result is in contrast to the findings of similar studies in developed countries which point to the positive impact of network connectivity on walking in older adults. However, it is supported by certain studies in other South American countries.

This finding on the association between network connectivity and the walking behavior of older adults offers a partial response to the second research question since it points to a major difference between developing and developed countries in terms of the walking behavior of older adults and the built environment factors that relate to it. Another major difference is that the majority of older adults, who walk in this context, are low-income adults. This is while in several developed countries a considerable percentage of the older adults who walk belong to high socio-economic status as well.

Finally, it is to be noted that since the secondary data was used as parts of the required data of this study—due to the limitations caused by COVID-19—the comparisons between the findings of this research and the new studies on walking behavior of older adults would contribute to having a more precise picture in terms of the association between their walking behavior and its relevant factors, as well as the changes of these associations before and after COVID-19 in this context. This is considered by the authors by defining the relevant studies in this context.

## Figures and Tables

**Table 1:** The summary measures regarding the built environment through both GIS and audit instruments.

Domain	Variable (Factor) *	Number of Items (In Audit Instrument)	Audit (Measured in Each Segment and Finally Calculated on the Scale of Each Buffer Zone)	Mean (In Total Zones) [SD]	GIS (Measured on the Scale of Each Buffer Zone)	Mean (In Total Zones) [SD]
**Density**						
	*Population density*				*Number of inhabitants per buffer*	321.55 [298.32]
	*Housing density*				*Number of housing units per buffer*	123.13 [101.91]
**Destinations (Accessibility and Diversity)**						
*Presence of destinations (Access to destinations)*						
	*Office/Institutional*	1	*1 = present, 0 = not present*	0.12 [0.4]		
	*Restaurant/Café/Commercial*	2	*1 = present, 0 = not present*	0.26 [0.44]		
	*Industrial*	3	*1 = present, 0 = not present*	0.09 [0.28]		
	*Vacant/Undeveloped*	4	*1 = present, 0 = not present*	0.08 [0.26]		
	*Recreation (Parks and plazas)* **	5	*1 = present, 0 = not present*	0.13 [0.33]	*Number of parks and plazas in each buffer*	0.79 [1.6]
	*Commercial*				*Numbers of commercial land uses per buffer*	5.93 [4.12]
	*Services*				*Number of services including bank and other types per buffer*	4.1 [5.56]
	*Education*				*Number of Educational destinations per buffer*	1.32 [1.63]
	*Health centers and hospitals*				*Number of Health centers and hospitals per buffer*	0.4 [0.73]
	*Access to public transport*				*total street length with access to public transport per area of each buffer zone*	9.80 [12.50]
*Diversity*						
	*Mix Land use* ***	1	*The proportion of segments with more than one destination present in each buffer*	0.27 [0.09]	*Entropy index (5 types of land uses)*	0.57 [0.12]
**Functionality (Design)**						
*Walkway’s structural features*						
	*Presence of pathway for pedestrian*	1	*1 = present, 0 = not present*	0.84 [0.42]		
	*Quality of pavement*	2	*1 = poor, 2 = fair, 3 = good*	2.73 [0.56]		
	*Track length*	3	*The real length of each segment (by step which later is transferred to meter; a single step = 0.762 m)*	154 [125.12]		
	*Sidewalk width*	4	*1 < 4 feet,* *2 = between 4 and 8 feet,* *3 > 8 feet*	1.13 [0.35]		
	*Physical barriers/path obstructions*	5	*1 = present, 0 = not present*	0.2 [0.58]		
	*The buffer between road and path*	6	*1 = present, 0 = not present*	0.85 [0.37]		
	*Path distance from curb*	7	*1 = at edge, 2 < 5 feet, 2 > 5 feet*	1.99 [0.60]		
	*Curb cuts*	8	*1 = none, 2 = 1–4, 3 > 4*	1.95 [0.26]		
	*Slope* **	9	*1 = flat, 2 = slight hill,* *3 = steep hill*	1.16 [0.48]	*1 = low slope (less than 5% slope), * *2 = medium slope (between 5–15% slope),* *3 = high slope (more than 15% slope)*	1.38 [0.72]
	*Amenities*	10	*1 = present, 0 = not present*	0.19 [0.42]		
*Street’s structural features*						
	*Wayfinding (Are there wayfinding aids?)*	1	*1 = no, 2 = yes*	1.58 [0.49]		
	*On-Street parking*	2	*1 = Parallel or Diagonal,* *2 = none*	1.53 [0.50]		
	*Off-street parking lot spaces*	3	*1 = no, 2 = 0–5, 3 = 6–25*	1.09 [0.30]		
	*Presence of Bicycle lanes (Are there bicycle lanes on the segment?)*	4	*1 = yes, 2 = no*	1.97 [0.16]		
*Permeability (Street connectivity)*						
	*Street connectivity/Sidewalk connectivity* ***	1	*Number of Sidewalk connections to other sidewalks/crosswalks*	4.83 [0.94]	*Link–Node Ratio (Links per unit of area (streets)/# Nodes per unit of area)*	1.54 [0.25]
					*Intersection Density (Real nodes area/Area)*	160.08 [123.6]
					*Street density (Total street length per unit of area/area)*	20.79 [18.54]
**Safety**						
*Traffic safety*						
	*Traffic control devices*	1	*1 = present, 0 = not present*	0.14 [0.35]		
	*Crossing aids*	2	*1 = present, 0 = not present*	0.24 [0.44]		
	*Posted speed limit*	3	*1 = present, 0 = not present*	0.06 [0.24]		
	*Crosswalks*	4	*1 = none, 2 = 1–2, 3 = 3–4,* *4 > 4*	1.15 [0.43]		
	*Presence of med-hi volume driveways*	5	*1 < 2, 2 = 2–4, 3 > 4*	1 [0.00]		
*Personal security*						
	*Surveillance (Visibility) (can be observed from a window, verandah, porch, garden)*	1	*1 = Can be observed from more than 75% of buildings* *2 = Can be observed from between 50–74% of buildings* *3 = Can be observed from less than 50% of buildings*	1.17 [0.39]		
	*Presence of people*	2	*Number of people in the segment*	1.62 [3.48]		
	*Roadway/path lighting*	3	*1 = road-oriented lighting,* *2 = pedestrian-scale lighting,* *3 = other lighting,* *4 = no lighting*	1 [0.00]		
**Aesthetic**						
*Streetscape*						
	*Number of trees* ***	1	*1 = non for very few, 2= some, 3= many/dense*	1.71 [0.50]	*Number of trees per buffer zone*	72.9 [87.6]
	*Overall cleanliness and building maintenance*	2	*1 = Poor (much litter/graffiti/broken facilities),* *2 = Fair (some litter/graffiti/broken facilities),* *3 = Good (no litter/graffiti/broken facilities)*	2.43 [0.62]		
	*Building height*	3	*1 = short, 2 = medium, 3 = tall*	1.09 [0.35]		
	*Articulation in building designs*	4	*1 = little or no articulation,* *2 = some articulation,* *3 = highly articulated*	1.27 [0.48]		
	*Public art (Is there public art that is visible in this segment?)*	5	*1 = yes, 2 = no*	2 [0.00]		
	*Degree of enclosure*	6	*1 = little or no enclosure,* *2 = some enclosure,* *3 = highly enclosed*	1.97 [0.21]		
	*The level of attractiveness for walking (The sector is attractive for walking)*	7	*1 = strongly agree,* *2 = agree,* *3 = disagree,* *4 = strongly disagree*	2.41 [0.67]		
*Views*						
	*Urban (houses and household gardens)*	1	*1 = present, 0 = not present*	0.82 [0.18]		
	*Commercial (shops, light industrial, offices, schools)*	2	*1 = present, 0 = not present*	0.18 [0.22]		
	*Nature (parks, community gardens where the level of care differs)*	3	*1 = present, 0 = not present*	0.09 [0.26]		

* The highlighted (Bold) factors (variables) are those that entered into the final regression model. ** The variables measured in both audit and GIS; and only one of them was entered into the final model of the regression analysis. *** The variables that were measured in both audit and GIS; and both were entered into the final model of regression analysis.

**Table 2:** Descriptive statistics of socio-demographic variables and familiarity (*N* = 463).

Variables	Variable Description	Frequency	Percentage	Mean	SD
Level of walking (Minutes per day)				15.87	12.32
*Socio-demographic variables and Familiarity*					
Age (Continuous)				71.89	9.53
Gender	0 = Female	294	63.5	0.37	0.482
	1 = Male	169	36.5		
Home owning situation	0 = Rented	65	14	0.86	0.348
	1 = Owner	396	85.5		
Education	Low (Primary school and Lower)	178	38.4		
	Intermediate (High School and similar degrees)	224	48.4		
	High (University degrees, bachelor’s and higher)	61	13.2		
Job situation	0 = Without a job or retired	338	73	0.27	0.444
	1 = With job	125	27		
Monthly income	0 = Medium to high income	100	21.6	0.78	0.412
	1 = Low income	363	78.4		
Access to Internet	0 = Without Internet	248	53.6	0.46	0.499
	1 = Have internet	215	46.4		
Access to TV	0 = No TV	234	50.5	0.49	0.500
	1 = Have TV	229	49.5		
Work at home	0 = No	436	94.1	0.06	0.235
	1 = Yes	27	5.9		
Driver’s license	0 = Do not Have	354	76.4	0.23	0.422
	1 = Have	109	23.6		
Time Living Years (Familiarity)	0 = More than one year	409	88.3	0.12	0.321
	1 = Less than one year	54	11.7		
Vehicles in each household	0 = Do not have	298	64.4	0.36	0.479
	1 = Have	165	35.6		
Number of Bicycles in each household				0.64	0.096
Number of People in each household				3.08	1.615
Number of total trips in each household				9.12	5.74
Number of total walking trips in each household on the day of travel registration				1.72	0.97

**Table 3:** The results of adjusted hierarchical multiple regression analysis for predicting walking behavior of older adults (*N* = 463).

Variables	Standardized Coefficients	t	*p*-Value	95% CI	VIF
*Socio-demographic variables and familiarity (Level 1)*					
Gender	0.156	2.844	0.005 **	1.23–6.75	1.43
Age	0.127	2.154	0.032 *	0.14–0.30	1.65
Home ownership situation	−0.078	−1.485	0.138	−6.38–0.89	1.29
Education (High education is the reference category)					
Dummy low education	−0.048	−0.515	0.607	−5.86–3.43	4.13
Dummy intermediate education	−0.019	−0.225	0.822	−4.50–3.57	3.30
Access to Internet	0.104	1.664	0.097	−0.46–5.62	1.86
Access to TV	−0.066	−1.138	0.256	−4.43–1.18	1.59
Job situation	0.075	1.226	0.221	−1.27–5.48	1.79
Monthly income	−0.005	−0.080	0.936	−3.81–3.51	1.81
Work at home	−0.004	−0.070	0.944	−5.80–5.40	1.39
Driver’s license	−0.043	−0.664	0.507	−4.97–2.46	1.94
Time Living Years (Familiarity)	−0.075	−1.390	0.165	−7.26–1.24	1.37
Vehicles in each household	−0.066	−1.103	0.271	−4.73–1.33	1.69
Number of Bicycles in each household	−0.004	−0.703	0.482	−1.95–0.92	1.50
Number of People in each household	0.086	1.161	0.247	−0.45–1.78	2.62
Number of total trips in each household	−0.151	−2.093	0.037 *	−0.62–−0.01	2.46
Number of total walking trips in each household	0.087	1.382	0.168	−0.45–2.59	1.88
*Variables of the built environment ^1^ (Level 2)*					
**Density**					
Housing density (GIS)	0.073	1.303	0.193	−0.02–0.14	1.50
**Destinations**					
***Access to destinations***					
Restaurant/Café/Commercial	0.030	0.311	0.756	−7.06–5.71	4.34
Vacant/Undeveloped	−0.043	−0.694	0.488	−7.70–6.55	1.81
Parks and plazas (GIS)	0.164	2.4227	0.016 *	0.33–3.20	2.15
Commercial (GIS)	0.037	0.451	0.652	−1.23– 5.78	3.13
Education (GIS)	−0.155	−2.409	0.017 *	−5.65–−0.34	1.95
Access to public transport (GIS)	−0.056	−0.989	0.323	−8.02–4.97	1.52
***Diversity***					
Mix land use	0.096	0.871	0.384	−1.57–4.77	4.74
Mix land use (GIS)	0.148	2.056	0.040 *	0.57–9.81	2.45
**Functionality (Design)**					
***Walkway’s structural features***					
Presence of pathway for pedestrian	0.120	1.902	0.058	−0.22–9.11	1.87
Sidewalk Width	0.082	1.643	0.101	−0.03–0.38	1.17
Quality of pavement	−0.097	−1.515	0.131	−9.21–1.97	1.94
Track length	0.140	2.198	0.029 *	0.39–6.03	1.91
Physical barriers/path obstructions	0.017	0.304	0.761	−3.73–4.10	1.48
The buffer between road and path	−0.004	−0.060	0.952	−4.61–4.22	1.96
Slope	0.111	1.529	0.127	−0.78–5.25	2.48
Amenities	−0.030	−0.516	0.606	−5.63–4.04	1.63
***Street’s structural features***					
On-Street parking	0.096	1.626	0.105	−0.89–5.47	1.65
Off-street parking lot spaces	−0.185	−2.671	0.008 **	−9.35–−2.93	2.28
***Permeability (Street connectivity)***					
Sidewalk connectivity	−0.079	−1.255	0.210	−3.54–0.78	1.88
Link Node Ratio (GIS)	−0.162	−2.293	0.022 *	−9.05–−1.22	2.35
Intersection Density (GIS)	0.018	0.228	0.820	−2.73–3.45	2.92
**Safety**					
***Traffic safety***					
Crossing aids	−0.046	−0.613	0.540	−4.78–3.55	2.66
Posted speed limit	0.088	1.528	0.127	−2.46–11.63	1.56
Traffic control devices	−0.068	−1.191	0.234	−5.67–2.37	1.55
***Personal security***					
Surveillance (Visibility)	0.058	0.943	0.346	−3.82–4.88	1.81
**Aesthetic**					
***Streetscape***					
Number of trees	−0.040	−0.604	0.546	−4.46–3.42	2.06
Number of trees (GIS)	0.087	1.368	0.168	−1.06–6.08	1.87
Overall cleanliness and building maintenance	−0.091	−1.325	0.186	−4.34–1.23	2.26
Building height	0.166	2.136	0.033 *	0.55–7.35	2.88
Articulations in building design	0.116	1.951	0.050 *	0.37–7.69	1.68
***Views***					
Nature	0.159	2.516	0.012 *	4.18–9.16	1.89

^1^ All the built environments were measured based on audit instruments and the variables measured by GIS were mentioned. * *p* < 0.05; ** *p* < 0.01. Dependent variable: Walking Behavior (minutes of walking per day); R Square: 0.240.

## Data Availability

Not applicable.
